# Isolation and Characterization of a Novel Phage against *Vibrio alginolyticus* Belonging to a New Genus

**DOI:** 10.3390/ijms25169132

**Published:** 2024-08-22

**Authors:** Jie Gao, Yuang Zhu, Rui Zhang, Juntian Xu, Runjie Zhou, Meiqi Di, Di Zhang, Wenxin Liang, Xing Zhou, Xing Ren, Huifang Li, Yunlan Yang

**Affiliations:** 1 Jiangsu Institute of Marine Resources Development, Jiangsu Ocean University, Lianyungang 222005, China; 2022220111@jou.edu.cn (J.G.); jtxu@jou.edu.cn (J.X.); 2020000044@jou.edu.cn (D.Z.);; 2 State Key Laboratory of Marine Environmental Science, College of Ocean and Earth Sciences, Xiamen University (Xiang’an), Xiamen 361005, China; 3 Institute for Advanced Study, Shenzhen University, Shenzhen 518061, China; ruizhang@szu.edu.cn; 4 State Key Laboratory of Trophic Oceanography, South China Sea Institute of Oceanology, Chinese Academy of Sciences, Guangzhou 510301, China; yc37948@um.edu.mo; 5 Centre for Regional Oceans, Department of Ocean Science and Technology, Faculty of Science and Technology, University of Macau, Macau 999078, China; 6 Guangxi Key Laboratory of Beibu Gulf Marine Resources, Environment and Sustainable Development/Ministry of Natural Resources, Beihai 536000, China

**Keywords:** *Vibrio alginolyticus*, phage, biological characterization, genomic analysis, phage therapy

## Abstract

*Vibrio alginolyticus* causes substantial economic losses in the aquaculture industry. With the rise of multidrug-resistant *Vibrio* strains, phages present a promising solution. Here, a novel lytic *Vibrio* phage, vB_ValC_RH2G (RH2G), that efficiently infects the pathogenic strain *V. alginolyticus* ATCC 17749^T^, was isolated from mixed wastewater from an aquatic market in Xiamen, China. Transmission electron microscopy revealed that RH2G has the morphology of Siphoviruses, featuring an icosahedral head (73 ± 2 nm diameter) and long noncontractile tail (142 ± 4 nm). A one-step growth experiment showed that RH2G had a short latent period (10 min) and a burst size of 48 phage particles per infected cell. Additionally, RH2G was highly species-specific and was relatively stable at 4–55 °C and pH 4–10. A genomic analysis showed that RH2G has a 116,749 bp double-stranded DNA genome with 43.76% GC content. The intergenomic similarity between the genome sequence of RH2G and other phages recorded in the GenBank database was below 38.8%, suggesting that RH2G represents a new genus. RH2G did not exhibit any virulence or resistance genes. Its rapid lysis capacity, lytic activity, environmental resilience, and genetic safety suggested that RH2G may be a safe candidate for phage therapy in combatting vibriosis in aquaculture settings.

## 1. Introduction

Vibriosis is generally referred to as a systemic bacterial infection, and considered a significant problem associated with severe economic losses in the aquaculture industry worldwide [1]. *Vibrio alginolyticus*, a Gram-negative bacterium, is characterized as moderately halophilic and mesophilic in nature [2]. Diseases caused by *V. alginolyticus* have resulted in mass mortalities among both vertebrates and invertebrates, causing considerable losses in aquaculture [3]. Strains of this bacterium have been identified as causative agents in vibriosis outbreaks affecting species like grouper (*Epinephelus malabaricus*) and sea bream (*Sparus aurata*) [4,5]. Over the past few decades, antibiotics have been heavily utilized in aquaculture for disease prevention and management, leading to the emergence of antibiotic-resistant bacteria [6]. There is a pressing need to reduce antibiotic use and explore alternative methods to control bacterial pathogens. 

Phages have been recognized as potential therapeutic agents for treating pathogenic *Vibrio* infections in aquaculture [7,8]. Trials of phage therapy on swimming crab *Portunus trituberculatus* larvae have shown that vB_Va_Valyong3 increased the survival rate of the larvae from 33.33% to 51.67% [9]. Additionally, the phage VA5 demonstrated significant inhibitory effects on *V. alginolyticus*-infected shrimp culture [10]. Previous studies have indicated that phage cocktails can effectively eliminate *Vibrio* species isolated from shrimp aquaculture environments [11]. With their high host-specificity, ability to proliferate, and minimal impact on surrounding microbial populations, phages have the potential to serve as potent and safe biocontrol agents [12]. 

In the realm of biocontrol applications, the selection of appropriate phages is a prerequisite. Prior to implementing phage therapy in real-world settings, rigorous testing must be conducted. Furthermore, a comprehensive analysis of the phage genome is imperative to ensure the absence of potentially harmful genes associated with virulence or antibiotic resistance. To date, 25 bacteriophages against *V. alginolyticus* have been isolated, including seven members with short, noncontractile tails (Vp670, AS51, ϕA318, PVA1, ϕV141, vB_ValP_IME234 and VEN) [13,14,15,16,17,18], six phages with contractile tails (VAP9, pVa-21, VAP21, ϕV208, ϕV172 and ValKK3) [16,19,20,21], and only three phages with long and noncontractile tails (ValSw3-3, VA5 and vB_Va_Val-yong3) [9,10,22] (Table S1). The remaining nine phages are still unidentified (Table S1). In this study, we reported the biological characteristics and genomic analysis of a novel bacteriophage, vB_ValC_RH2G, that infects *V. alginolyticus* ATCC 17749^T^. Our examination encompassed the phage’s morphology, host specificity, infection kinetics, lytic cycle, stability, and genetic profile, with the aim of assessing its suitability for future phage therapy applications. 

## 2. Results

### 2.1. Biological Characterization of RH2G

**Figure 1:**
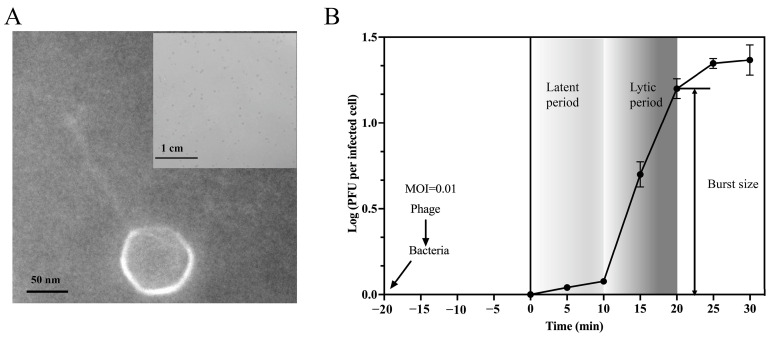
Isolation and growth curve of vB_ValC_RH2G. (**A**) Transmission electron micrograph of vB_ValC_RH2G. (**B**) One-step growth curve of vB_ValC_RH2G. Error bars indicate standard deviations among triplicate samples.

By propagating vB_ValC_RH2G (hereafter RH2G) on its host strain, *V. alginolyticus* ATCC 17749^T^, plaques were formed on a bacterial lawn with a clear round morphology after 24 h of incubation at 28 °C (Figure 1A). Observation by transmission electron microscopy revealed that RH2G possessed an icosahedral head (73 ± 2 nm) and a long, noncontractile tail (142 ± 4 nm) (Figure 1A). It belongs to the Siphovirus morphotype. 

Among 14 strains of *Vibrio*, spot tests showed that RH2G only infected the original host, *V. alginolyticus* ATCC 17749^T^ (Table 1). The results showed that RH2G has a high degree of specificity for its host strain, and is likely a strain-specific phage. Furthermore, phage RH2G exhibited resistance to all three concentrations of chloroform test (0 µL, 20 µL and 200 µL of chloroform), indicating an absence of lipids within its viral capsid.

The one-step growth curve was constructed to understand the lytic cycle of RH2G (Figure 1B). The latent period, defined as the minimum time it takes from phage adsorption to lysis of the host with the release of progeny virions, was determined to be 10 min. The burst size, denoting the average number of progeny virions liberated by one infected host–cell at the completion of a growth cycle of RH2G, was estimated to be around 48 plaque-forming units (PFU cell^−1^). 

### 2.2. Lytic Ability of vB_ValC_RH2G

**Figure 2:**
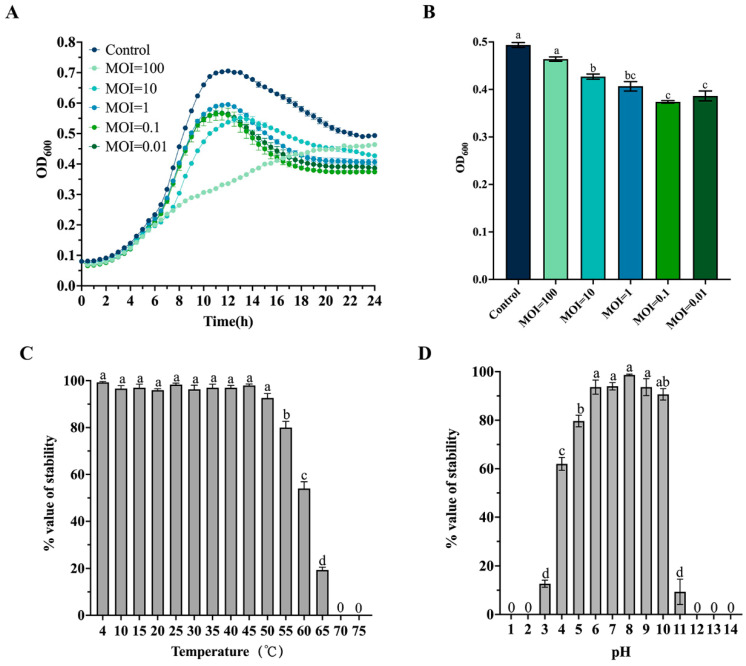
Biological features of phage vB_ValC_RH2G. (**A**) Growth curve for *V. alginolyticus* ATCC 17749T infected by vB_ValC_RH2G. (**B**) Absorbance (OD600) of the host at 24 h. (**C**) Thermal stability profile. (**D**) pH stability profile. Error bars indicate standard deviations among triplicate samples. Letters on the columns indicate statistical significance at *p* < 0.05.

The killing curve showed that phages at multiplicities of infection (MOIs) of 0.01, 0.1, and 1 did not significantly inhibit bacterial growth within the first 10 h, then showed a significant inhibitory effect (Figure 2A). Conversely, RH2G showed the most inhibitory effect at an MOI of 100 within 7–16 h. Overall, RH2G at MOIs of 0.01, 0.1, 1, and 10 significantly inhibited host growth compared to the control after 24 h of incubation (*p* < 0.001) (Figure 2B). 

### 2.3. Thermal and pH Stability of RH2G

To assess the stability of RH2G under different environmental conditions, we exposed it to various temperature and pH values, and quantified the changes in PFUs. The phage remained stable between 4 °C and 50 °C after 1 h of treatment, while the infectivity declined to approximately 80% at 55 °C and 54% at 60 °C. Above 65 °C, the phage retained less than 20% of its infectivity (Figure 2C). Moreover, stability testing demonstrated that RH2G remained stable over a broad pH range, from 6.0 to 10.0 (Figure 2D). Even at pH 4–5, the infectivity remained above 60%, dropping below 20% only when the pH was below 4 or above 11.

### 2.4. Genomic Characterization and Taxonomy of RH2G

**Figure 3:**
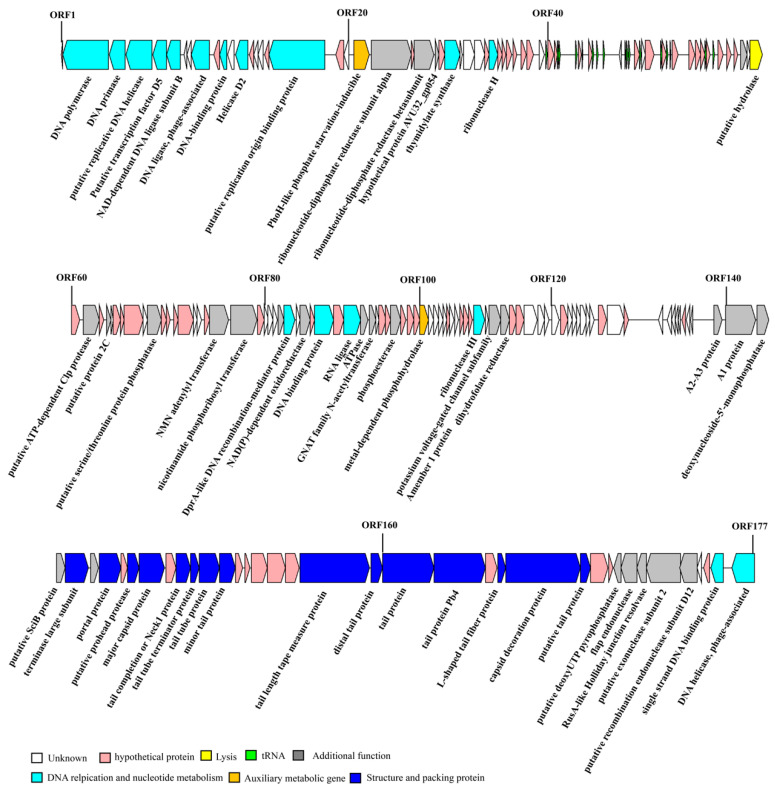
Genome map of the *V. alginolyticus* phage vB_ValC_RH2G. Protein-coding sequences are presented by arrows. Gene features classified into different functional modules are color-coded according to the legend below the figure.

The genome of RH2G was sequenced using the Illumina MiSeq platform. Following de novo assembly, a linear 116,749 bp double-stranded DNA genome with a G+C content of 43.76% was obtained. An analysis of the termini revealed that RH2G utilizes a headful (pac) packaging mechanism and contains redundant ends for circularizing the phage genome by recombination (Figure S1). The genome contained 177 open reading frames (ORFs) and 17 tRNAs (Figure 3, Table 2). Roughly 34.46% (61 ORFs) of the ORFs were related to functional proteins while another 38.42% were annotated as proteins with hypothetical functions (Table S2). A BlastP analysis indicated that 69 ORFs share similarity with the *Vibrio* phage VCPH (ranging from 33.33% to 84.38%), 15 ORFs with the *Vibrio* phage vB_VpS_PG07 (34.18–90.83%), 12 ORFs with the *Vibrio* phage vB_ValS_X1 (44.59–97.33%), and 9 ORFs with the *Vibrio* phage VspSw_1 (44.71–79.56%). The functional ORFs were categorized into five functional modules: phage structure and packaging, phage lysis, DNA replication and metabolism, auxiliary metabolic genes (AMGs), and additional functions (Figure 3, Table S2). The phage structure and packaging proteins group encompassed 15 ORFs, with 14 encoding typical structural proteins, such as the major capsid protein (ORF 148), portal protein (ORF 145), and tail-related proteins (ORF 147, ORF 150, ORF 151, ORF 152, ORF 153, ORF 159, ORF 160, ORF 161, ORF 162, ORF 164, ORF 165 and ORF 166), along with one gene encoding the terminase large subunit (ORF 143) of the phage packaging protein. Seventeen functional genes were related to DNA replication and nucleotide metabolism, including DNA polymerase (ORF 2), DNA primase (ORF 3), DNA helicase-related protein (ORF 4, ORF 13, ORF 177), DNA binding-related protein (ORF 11, ORF 18, ORF 88, ORF 176), ligase-related protein (ORF 6, ORF 9, ORF 90), DprA-like DNA recombination-mediator protein (ORF 84), RNA ligase (ORF 90), ribonuclease-related protein (ORF 32, ORF 111), thymidylate synthase (ORF 27), and transcription factor D5 (ORF 5). The lysis-related gene was putative hydrolase (ORF 59). Furthermore, two genes were identified as AMGs, including ORF 21 (*PhoH*-like phosphate starvation-inducible) and ORF 100 (metal-dependent phosphohydrolase). Twenty-six ORFs were attributed with additional functions (Table S2). The PhageAI analysis indicated the 100% probability of RH2G’s virulence. No virulence or resistance genes were detected in RH2G.

**Figure 4:**
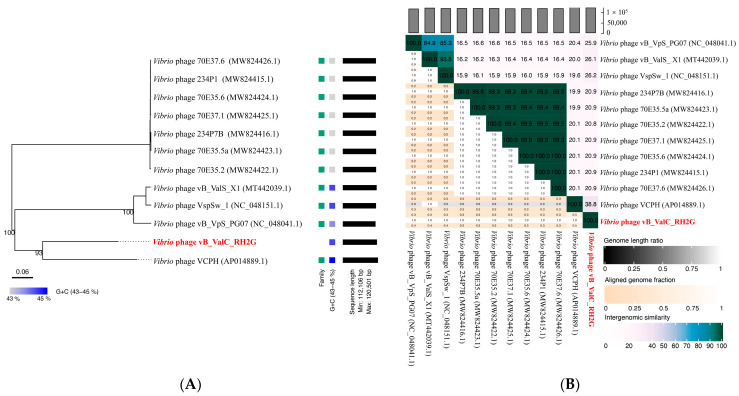
Taxonomy and phylogeny of vB_ValC_RH2G. (**A**) Phylogenetic tree of vB_ValC_RH2G and other closely related phages, constructed using the Virus Classification and Tree Building Online Resource (VICTOR) web service. Pairwise comparisons of the amino acid sequences were conducted using the Genome-BLAST Distance Phylogeny (GBDP) method with settings recommended for prokaryotic viruses. (**B**) Pairwise intergenomic distances/similarities among viral genomes according to the Virus Intergenomic Distance Calculator. Intergenomic similarity values are in right half, alignment indicators are in left half and top annotation.

**Figure 5:**
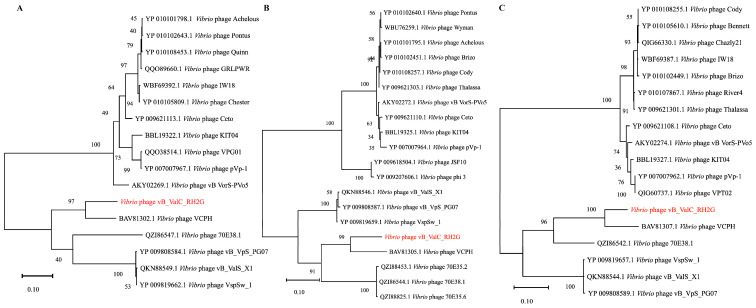
Maximum-likelihood phylogenetic tree (1000 bootstraps) based on amino acid sequences of (**A**) major capsid protein, (**B**) portal protein, and (**C**) terminase large subunit.

Our analysis revealed that the genome of RH2G exhibited genetic similarity to other *Vibrio* phage genomes in the NCBI database, with identities ranging from 97.1% to 76.7% and little coverage (8–14%), i.e., *Vibrio* phage vB_VpS_PG07 (accession number NC_048041.1), *Vibrio* phage VCPH (accession number AP014889.1), and *Vibrio* phage VspSw_1 (accession number NC_048151.1). This suggested that phage RH2G differs significantly from other phages in the NCBI database, indicating the isolate represents a novel *V. alginolyticus* phage. A phylogenetic tree was constructed based on pairwise comparisons of the amino acid sequences of RH2G and related phages detected by VIPTree confirmed that phage RH2G and VCPH formed a separated clade (Figure S2 and Figure 4A). Additionally, the phylogenetic tree based on the major capsid protein (MCP), portal protein, and terminase large subunit protein (TerL) further supported the close relationship between RH2G and the *Vibrio* phage VCPH (Figure 5). The VIRIDIC results indicated a maximum intergenomic similarity of 38.8% between RH2G and the *Vibrio* phage VCPH, falling below the 70% similarity thresholds for genus classification (Figure 4B) [23]. Therefore, RH2G was considered to belong to a new genus. 

## 3. Discussion

In this study, a novel *V. alginolyticus* phage named RH2G was isolated and characterized, belonging to a new genus. The burst size and latent period of RH2G were 48 PFU/cell and 10 min, respectively. Notably, phages infected *V. alginolyticus* have been observed to have short latent periods (<30 min) and small burst sizes (ranging from 10 to 100 PFU/cell) such as ValSw3-3 (95 PFU/cell, 15 min), VA5 (92 PFU/cell, 20 min), and vB_Va_Val-yong3 (87 PFU/cell, 30 min) [9,10,22]. For lytic phages, there exists a trade-off between the burst size and latent period. This is because the release of phages from infected cells comes at the expense of the cellular machinery required to produce additional phage progeny. [24]. Phages can achieve faster population growth through reductions in the latent period despite a decrease in burst size; those with very short latent periods at the expense of burst size may be seen as specialists for propagation in high-bacterial-density environments. A shorter latent period was thought to represent a specialization for the exploitation of bacteria growing at higher densities, which can substantially reduce phage generation times. 

The tail length in Siphophages is determined by the tap measure protein (TMP) and is directly proportional to the size of the TMP, with one amino acid of the TMP corresponding to 0.145 nm of tail length [25]. Using this equation, the expected tail length of RH2G was 138 nm (TMP consisting of 950 amino acids), aligning with the electron micrograph estimations of 142 ± 4 nm (Figure 1B). This high specificity is advantageous as it allows phages to target specific bacteria without harming natural microorganisms, especially in environments where specific Vibrio strains are prevalent. Interactions between the tip of long tail fibers and lipopolysaccharides play a crucial role in host recognition for tailed phages. The specific shape and size of its tip give RH2G distinct specific receptor-binding properties [26]. This high specificity is advantageous as it allows phages to target specific bacteria without harming natural microorganisms during treatment, especially in environments where specific *Vibrio* strains are prevalent. Moreover, this phage can also be combined with other phages to create a phage cocktail, effectively targeting a broader spectrum of bacteria and reducing the development of resistance.

Numerous genes related to the replication and regulation of phage DNA are predominantly located in the upstream region of the RH2G genome. For instance, ORF 2 encodes DNA polymerase and ORF 3 encodes DNA primase, responsible for synthesizing short oligonucleotides, typically RNA, serving as primers to facilitate DNA polymerization. ORF 9 encodes DNA ligases, which can join 3′-OH and 5′-PO4 termini to form a phosphodiester, being essential for DNA replication and repair. The presence of these genes suggests that RH2G is capable of independently replicating its DNA within the host. Single-strand DNA binding proteins (SSBs) perform critical functions in genome maintenance including DNA replication, recombination, and repair. They bind with high affinity to ssDNA in a non-sequence-specific way, protect ssDNA from degradation, prevent the formation of secondary structures, and interact with a plethora of proteins involved in DNA metabolism, recruiting them to their sites of action and stimulating their activities [27]. Some viruses rely on the host SSBs; for example, phage λ uses an *Escherichia coli* [28] SSB for replicating their genome. ORF 176 in the RH2G genome was predicted to encode an SSB, which is similar to the gp32 of the *Escherichia* phage RB69 that contains three function domains: a core ssDNA binding domain that harbors the OB-fold, an N-terminal domain involved in cooperative interactions, and the acidic C-terminal domain that mediates interactions with the replisome proteins [29]. ORF 177 encodes phage DNA helicase, which is essential for DNA replication, expression, recombination, and repair through the ATP-dependent unwinding of dsDNA.

Two genes identified as AMGs in RH2G are ORF 21 (*PhoH*-like phosphate starvation-inducible) and ORF 100 (metal-dependent phosphohydrolase). The AMGs are phage-encoded and host-derived metabolic genes that are presumed to be involved in regulating host metabolism to enhance viral replication. The *phoH* gene is part of the Pho regulon, responsible for regulating phosphate uptake and metabolism in conditions of low phosphate levels and phosphate limitation. Phosphorus is a crucial element necessary for nucleotide biosynthesis and DNA replication. This gene could be upregulated in response to phosphate starvation in host cells, with its products playing a vital role in regulating phosphorus absorption and transportation in host cells under conditions of low phosphorus content or deprivation [30]. The frequent occurrence of *phoH* genes in phage genomes suggests that their products play a role in the phosphate metabolism of the phage-infected cell [31]. Research has also demonstrated that cyanophages maintain the *phoH* gene to enhance their host’s phosphate uptake during infection, though the exact mechanism is not fully understood [1]. The *phoH* gene has also been reported in *Vibrio* phages, such as phage KVP40, a well-studied T4-like phage isolated from polluted coastal sea water in Japan [32]. The presence of genes involved in phosphorous acquisition in RH2G suggests that this phage has developed adaptations to thrive in oligotrophic environments [33]. RH2G contains an HD domain-containing hydrolase-like enzyme encoded by ORF 100, which exhibits dATPase activity. It can catalyze the hydrolysis of dATP to dA and triphosphate, as well as the hydrolysis of dADP and dAMP into dA, releasing pyrophosphate and phosphate [34]. The dATPase facilitates Diaminopurine genome synthesis by specifically removing dATP and its precursor dADP from the host’s nucleotide pool, preventing the incorporation of adenine into the phage genome. The diaminopurine genome-biosynthetic system, consisting of dATPase, DNA polymerase, and diaminopurine synthetase, helps evade host restriction enzyme attacks [35]. It is worth noting that no genes related to diaminopurine synthetase were found in the RH2G genome.

The phage’s efficiency in bacterial inactivation, a critical property for phage therapy candidates, was evaluated through phage lysis tests. RH2G exhibited inefficacy in eliminating the host in the first seven hours at every MOI value, possibly due to a lack of phages capable of lysing a substantial amount of *Vibrio* in a short period. MOIs of 0.1 and 0.01 showed the most effective lysis of the host cells, while compared with MOIs of 0.1 and 0.01, the host bacteria showed resistance early at the MOI of 100. Thus, appropriate phage dosing is essential for effective phage therapy applications. Furthermore, RH2G maintained high lytic activity across diverse environmental conditions (4–55 °C and pH 4–10). RH2G showed a greater acid tolerance than vB_ValP_IME234 (pH 6–10), VAP9 (pH 6–8), and VAP21 (pH 7–11) [19], demonstrating that RH2G is more stable and exerts bactericidal activity in practical applications, suggesting that it is a promising candidate for the biological control of *V. alginolyticus*.

The biological and genomic characteristics are critical in evaluating phage fitness and identifying candidates for use in phage therapy. The host specificity, life cycle parameters, and stability are the main prerequisites that need to be considered. Our fundings demonstrated that the narrow host range of RH2G would enable its use to specifically target *V. alginolyticus*. Furthermore, phages with narrow host ranges can be added to phage cocktails to combat a wide range of bacteria and reduce the development of resistance. The shorter latent period for RH2G (10 min) was beneficial for quickly suppressing bacterial proliferation when the bacterial density was sufficiently high. RH2G demonstrated high lytic activity across diverse environmental conditions (ranging from 4 °C to 55 °C and pHs levels 4 to 10), maintaining stability values of 20.1%, 12.7%, and 10.3% at 65 °C, pH 3, and pH 11, respectively. Regarding the genomic characteristics, no virulence or resistance genes were detected in RH2G, indicating that it is unlikely to lead to the enhancement of *vibrio* virulence or the contamination of antibiotic resistance genes when applied in aquaculture environments. Moreover, the PhageAI analysis indicated a 100% probability of RH2G’s virulence. Therefore, RH2G might be more stable, safer, and exert bactericidal activity in practical applications. 

## 4. Materials and Methods

### 4.1. Isolation and Purification of Phages

The host strain used in this study, *V. alginolyticus* ATCC 17749^T^, was purchased from the China General Microbial Culture Collection Center (CGMCC) in January 2019. The phage-containing water was collected from mixed wastewater from aquaculture at Xia Shang aquatic market in Xiamen, China, and filtered through a 0.22 μm pore size filter membrane (Millipore, Bedford, MA, USA) [26]. After enrichment, the isolation of phage plaques was by gradient dilution and the double-layer agar plate method, as described by Clokie et al. [36]. Phages were purified by selecting a single plaque, dissolving in SM Buffer (100 mM NaCl, 8 mM MgSO_4_, 5 mM Tris-HCl (pH 7.5), 2% gelatin), and purifying through at least three repetitions.

### 4.2. Host Range Detection and Chloroform Sensitivity Testing of Phage

The host range of the RH2G was assessed using a spot assay and validated with the double-layer agar method. Apart from *V. alginolyticus* ATCC 17749^T^, 13 *Vibrio* strains were used in the host range assessment. Each exponentially growing bacterial culture was combined with molten soft agar (0.5% [wt/vol]), then immediately poured onto a solid agar plate (1.5% [wt/vol]). Once the agarose plates solidified, 5 µL of phage lysate was spotted onto the bacterial lawn. The agar plates were then incubated for >24 h at 28 °C and examined for the presence of a lysis zone to ascertain phage infection of the host bacteria. This experiment was repeated three times. *V. alginolyticus* 17749^T^ served as the positive control, while SM buffer was the negative control.

### 4.3. Transmission Electron Microscopy

The purified phages were negatively strained with phosphotungstic acid (1%, wt/vol, pH 7.2). Transmission electron microscopy (TEM) (JEOL JEM-1200EX; JEOL, Tokyo, Japan) operating at 80 kV was employed to capture images of the purified RH2G phage particles [37]. Images were recorded using the CCD image transmission system (Gatan Inc., Pleasanton, CA, USA). Phage size was measured using ImageJ v2.35 (http://imagej.net/ [accessed on 1 April 2024]) based on at least five individual phage particles [38].

### 4.4. One-Step Growth Curve Determination

To assess the infectivity and replication ability of RH2G, the one-step growth curve method was employed to determine the burst size and latent period [38]. Briefly, the bacterial culture in exponential growth phase was mixed with 1 mL of phage to produce a multiplicity of infection (MOI) of 0.1. The unabsorbed phage particles were removed by centrifugation (10,000× *g* for 10 min) [39]. Samples were then taken every 5 min over a span of 100 min, with three biological replicates and analyzed using the double-layer agar method [40]. 

### 4.5. Thermal and pH Stability

The purified phage solution was statically incubated for 1 h at various temperature gradients (4–75 °C). For pH stability assessment, phage samples were incubated into SM buffer at pH values from 1 to 14, and statically incubated for 1 h at 25 °C. The phage titer, displaying thermal and pH stability, was determined using the double-layer plate method and conducted in triplicate. All experiments were performed at three times. SM buffer was the negative control. Statistical differences were determined using one-way analysis of variance followed by Duncan’s multiple range test at *p* < 0.05 [37]. 

### 4.6. DNA Extraction, Genome Sequencing, and Phylogenetic Analysis 

Phage DNA was obtained using phenol–chloroform extraction [41]. The phage DNA was dissolved in 100 µL TE buffer (10 mM Tris-HCl, 1 mM EDTA, pH 8.0) and sequenced using Illumina platform with a 150 bp paired-end DNA library. Velvet software (v1.2.03) was utilized for genome assembly after removing low-quality reads [42]. Phage termini and packaging mechanisms were predicted with the PhageTerm tool (v3.0.1) [43]. RAST online server (http://rast.nmpdr.org [accessed on 24 March 2024]) was used to identify ORFs [44]. Nucleotide and protein sequences were scanned for homologs using BLAST (http://blast.ncbi.nlm.nih.gov/, database updated on 25 March 2024) [45] and an HHpred (https://toolkit.tuebingen.mpg.de/hhpred [accessed on 4 April 2024]) search [46]. The Eeayfig tool (v2.2.2) was used for genome visualization [31]. tRNAScan-SE v2.0 (http://lowelab.ucsc.edu/tRNAscan-SE/ [accessed on 4 April 2024]) was used to search for tRNA genes [47]. A genome-based life cycle classification was performed using an AI-driven software platform (https://phage.ai/ [accessed on 14 August 2023]). The absence of potentially toxic genes and antibiotic resistance was checked using virulence factors of pathogenic bacteria and a comprehensive antibiotic resistance database [23,48].

Intergenomic similarities between the phage and related phages were determined based on nucleotide data using the Virus Intergenomic Distance Calculator [23]. A proteomic tree, based on the whole-genome amino acid sequences and viral conserved proteins (MCP, TerL, and portal protein) of RH2G and *vibrio* phages, was generated using VipTree (http://www.genome.jp/viptree [accessed on 18 April 2024]) and MEGA (v11). The tBLASTx algorithm in VipTree (http://www.genome.jp/viptree [accessed on 18 April 2024]) was used to perform the genome comparisons between RH2G and its closest relatives. 

Details on the processing method are supplied in the Supporting Information.

## Figures and Tables

**Table 1:** Host range of phage vB_ValC_RH2G (+: infected; −: uninfected).

Species	Strain	Accession No.	Infectivity
*V. alginolyticus*	ATCC 17749^T^	NR_121709.1	+
*V. alginolyticus*	GS_MYPK1	NR_113609.1	−
*V. anguillarum*	NBRC 13266	JX684108.1	−
*V. anguillarum*	SE2011	CP031527.1	−
*V. anguillarum*	Ba35-E2-R3	MT876113.1	−
*V. anguillarum*	BVA1	MT860356.1	−
*V. crassostreae*	2-1	MT796337.1	−
*V. crassostreae*	20-1-16	MK102596.1	−
*V. crassostreae*	B22-2	MG867506.1	−
*V. crassostreae*	201709CJKOP-55	NR_044078.1	−
*V. crassostreae*	LGP 7	MG554497.1	−
*V. hangzhouensis*	G11	NR_114630.1	−
*V. parahaemolyticus*	ATCC 17802	CP034305.1	−
*V. parahaemolyticus*	20151116002-3	CP054700.1	−

**Table 2:** Summary of tRNAs in phage vB_ValC_RH2G.

tRNA Gene	Positions	Attribute	Anticodon
1	26,705–26,779	Lys	TTT
2	27,332–27,403	Glu	TTC
3	27,414–27,485	Glu	TTC
4	28,340–28,414	Asn	GTT
5	28,685–28,760	Asp	GTC
6	29,253–29,325	Ile	GAT
7	29,487–29,561	Pro	TGG
8	29,854–29,927	Gln	TTG
9	31,045–31,119	Leu	TAG
10	31,160–31,234	Leu	TAA
11	31,244–31,337	Ser	TGA
12	31,870–31,941	Thr	TGT
13	33,013–33,085	Val	TAC
14	33,338–33,411	Met	CAT
15	33,521–33,594	Phe	GAA
16	35,467–35,540	Cys	GCA
17	35,900–35,973	His	GTG

## Data Availability

The genome of *V. alginolyticus* phage vB_ValC_RH2G was deposited in GenBank under accession number PP034752.

## Supplementary Materials

The following supporting information can be downloaded at: https://www.mdpi.com/article/10.3390/ijms25169132/s1. References [9,13,14,15,16,17,18,19,20,21,22,23,26,31,36,37,38,39,40,41,42,43,44,45,46,47,48,49,50,51,52,53,54,55] are cited in the supplementary materials.
