# Supplementary material for: Isolation and Characterization of a Novel Phage against Vibrio alginolyticus Belonging to a New Genus
Source: Int J Mol Sci. 2024 Aug 22;25(16):9132. doi: 10.3390/ijms25169132 (PMC11354583; doi:10.3390/ijms25169132)
Supplement: Supplementary file 1 [file ijms-25-09132-s001.zip › phage vB_ValC_RH2G-Supplementary Materials-resubmitted.pdf]

Supplementary Information for

Isolation and characterization of a novel phage belonging to a new genus against *Vibrio alginolyticus*

Jie Gao[14], Yuang Zhu[1], Rui Zhang[24], Juntian Xu[1], Runjie Zhou[56], Meiqi Di[1], Di Zhang[13], Wenxin Liang[1], Xing Zhou[1], Xing Ren[3], Huifang Li[14*], Yunlan Yang[2*]

[1] Jiangsu Institute of Marine Resources Development, Jiangsu Ocean University, Lianyungang 222005, Jiangsu, China

[2] Institute for Advanced Study, Shenzhen University, Shenzhen 518061, Guangdong, China

[3] Guangxi Key Laboratory of Beibu Gulf Marine Resources, Environment and Sustainable Development/Ministry of Natural Resources, Beihai 536000, Guangxi, China

[4] State Key Laboratory of Marine Environmental Science, College of Ocean and Earth Sciences, Xiamen University (Xiang'an), Xiamen 361005, Fujian, China.

[5] State Key Laboratory of Trophic Oceanography, South China Sea Institute of Oceanology, Chinese Academy of Sciences, Guangzhou 510301, Guangdong, China.

[6] Centre for Regional Oceans, Department of Ocean Science and Technology, Faculty of Science and Technology, University of Macau, Macau 999078, China

*Correspondence

E-mail: Huifang Li: huifangli@jou.edu.cn

Yunlan Yang: yangyunlan@szu.edu.cn

This file includes:

Methods

Figure S1-S2

Tables S1-S2

Methods

Isolation and purification of phage

The host strain of *V. alginolyticus* ATCC 17749^T used in this study was purchased from the China General Microbial Culture Collection Center (CGMCC) in January 2019. The phage-containing water was collected from the mixed wastewater from aquaculture at Xia Shang aquatic market in Xiamen, China, and filtered through 0.22 μm pore size filter membrane (Millipore, Bedford, MA, USA) [1]. To increase the probability of phage isolation, the filtered phage incubated with exponentially growing host culture overnight at 28°C with shaking. Following incubation, the mixed culture was underwent centrifugation at 8000 × g for 10 min at 4°C and the supernatant was then filtered using 0.22 μm sterile filters to remove residual host cells and impurities. After enrichment, the isolation of phage plaques was by gradient dilution and the double-layer agar plate method as described by Clokie et al [2]. Specifically, 100 μL of phage mixed with 300 μL of indicator bacterium was added to 5 mL of RO medium containing 0.5% agar. This mixture was subsequently poured onto a prepared RO medium supplemented with 1.5% agar. After overnight incubation at 28°C, the phage plaques that appeared on the double-layer plates were enumerated. Phage were purified by selecting a single plaque, dissolved in SM Buffer (100 mM NaCl, 8 mM $MgSO_4$, 5 mM Tris-HCl (pH 7.5), 2% gelatin) and purified through at least three repetitions [3].

Host range detection and chloroform sensitivity testing of phage

The host range of the RH2G was assessed using a spot assay and validated with the double-layer agar method. Apart from *V. alginolyticus* ATCC 17749^T, 13 *Vibrio* strains were used in the host range assessment. Each exponentially growing bacterial culture was combined with molten soft agar (0.5% [wt/vol]), then immediately poured onto a solid agar plate (1.5% [wt/vol]). Once the agarose plates solidified, 5 μL of phage lysate was spotted onto the bacterial lawn. The agar plates were then incubated for >24 h at 28°C and examined for the presence of a lysis zone to ascertain phage infection of the host bacterium. This experiment was repeated three times. *V. alginolyticus* 17749^T served as the positive control, while SM buffer was the negative control [4].

Transmission electron microscopy

The purified phages were negatively strained with phosphotungstic acid (1%, wt/vol, pH 7.2). Transmission electron microscopy (TEM) (JEOL JEM-1200EX; JEOL, Japan) operating at 80 kV was employed to capture images of the purified phage RH2G particles [5]. Images were recorded

using the CCD image transmission system (Gatan Inc., Pleasanton, CA, USA). Phage size were measured using ImageJ v2.35 (http://imagej.net/) based on at least five individual phage particles [6].

One-step growth curve determination

To assess the infectivity and replication ability of RH2G, the one-step growth curve method employed to determine the burst size (the average number of phage particles that a single infected bacterium can produce) and latent period (the time interval between absorption and the beginning of the first burst) [6]. Briefly, the bacterial culture in exponential growth phase was mixed with 1 mL of phage to produce a multiplicity of infection (MOI) of 0.1. the unabsorbed phage particles were removed by centrifugation (10000 × g for 10 min) [7]. Samples were then taken every 5 min over a span of 100 min, with three biological replicates and analyzed using the double-layer agar method [8,9].

Thermal and pH stability

Thermal and pH stability tests were conducted to evaluate the impact of environmental factors on RH2G. The purified phage solution (10^8 PFU/mL) was statically incubated for 1 h at various temperature gradients (4°C, 10°C, 15°C, 20°C, 25°C, 30°C, 35°C, 40°C, 45°C, 50°C, 55°C, 60°C, 65°C, 70°C and 75°C). For pH stability assessment, phage sample were incubated into SM buffer at pH values from 1 to 14), achieved by adjustment with 1M NaOH and 1M HCL, and statically incubated for 1 h at 25°C. The phage titer of thermal and pH stability was determined using the double-layer plate method and conducted in triplicate. All experiments were performed at three times[10]. SM buffer was the negative control. Statistical differences were determined using one-way analysis of variance followed by Duncan's multiple range test at $p < 0.05$ [5].

DNA extraction

Phage DNA was achieved using phenol-chloroform extraction [11]. Following treatment with proteinase K (100 mg/mL), EDTA (0.5 mol/L), and 10% (w/v) SDS at 55°C, for 3 h, the digested sample underwent purification with phenol/chloroform/isoamyl alcohol (25:24:1) and chloroform/isoamyl alcohol (24:1) to remove any debris. Subsequently, the DNA pellet was sequentially precipitated with isopropanol and stored overnight at − 20°C, then washed twice with cold 70% ethanol and air-dried at room temperature. Finally, the phage DNA was dissolved in 100 μL TE buffer (10 mM Tris-HCl, 1 mM EDTA, pH 8.0) and stored at − 80°C before sequencing[12].

Genome sequencing and annotation

The phage genome was sequenced using Illumina platform with a 150-bp paired-end DNA library. Velvet software (v1.2.03) was utilized for genome assembly after removing low-quality reads [13]. Phage termini and packaging mechanisms were predicted with the PhageTerm tool (v3.0.1) [14]. RAST online server (http://rast.nmpdr.org [accessed on 24 March 2024]) was used to identified putative open reading frame (ORF) [15]. Nucleotide and protein sequences were scanned for homologs using BLAST (http://blast.ncbi.nlm.nih.gov/, database updated on 25 March 2024) [16] and an HHpred search was performed using the online server (https://toolkit.tuebingen.mpg.de/hhpred [accessed on 4 April 2024]) (E-value $\leqslant 10^{-3}$) [17]. The Eeayfig tool was used for genome visualization [18]. tRNAScan-SE v2.0 (http://lowelab.ucsc.edu/tRNAscan-SE/ [accessed on 4 April 2024]) was used to search for tRNA genes [19]. A genome-based life cycle classification was performed using an AI-driven software platform (https://phage.ai/ [accessed on 14 August 2023]). The absence of potentially toxic genes and antibiotic-resistance was checked using virulence factors of pathogenic bacteria and comprehensive antibiotic resistance database [20,21].

Phylogenetic analysis and comparative genomic analyses

Intergenomic similarities between the phage and related phages were determined based on nucleotide data using the Virus Intergenomic Distance Calculator [20]. The proteomic tree, based on the whole-genome amino acid sequences of phage RH2G and vibrio phages, was generated using VipTree (http://www.genome.jp/viptree [accessed on 18 April 2024]). tBLASTx and VipTree were used to perform the genome comparisons between RH2G and its closest relatives. Viral conserved proteins (MCP, TerL, and portal protein) were constructed to evaluate the evolutionary relationships among RH2G and other diverse phages, using MEGA.11 with a bootstrap of 1000.

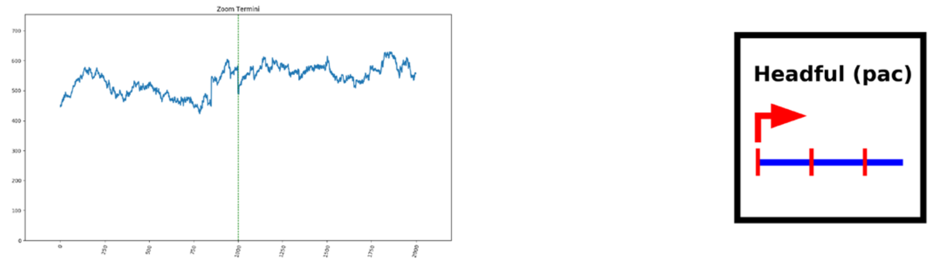

Figure S1 Termini Analysis of vB_ValC_RH2G by Phageterm.

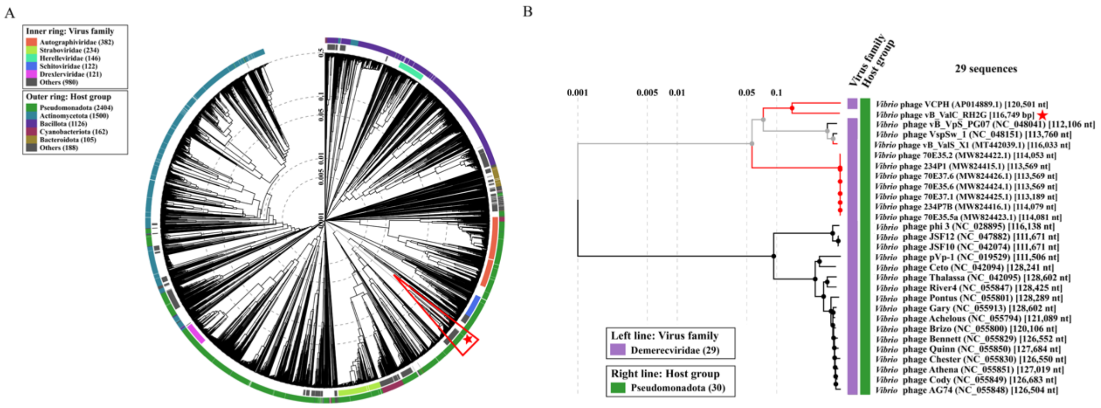

Figure S2 Proteomic tree analysis. (A) Proteomic tree analysis of 3985 phages. (B) Proteomic tree analysis of vB_ValC_RH2G with other 28 *Vibrio* phages. Phage vB_ValC_RH2G was labeled with a red star respectively.

Table S1 Isolated bacteriophages against *Vibrio alginolyticus*

Phage	Host	Morphology			Genome		One-step curve		Stability		Reference
		Category	Capsid diameter (nm)	Tail length (nm)	Size (bp)	ORFs	Latent period (min)	Burst size (PFU/cell)	Temperature (℃)	pH	
Vp670	E06333	Podoviridae	55	15	43,121	49	30	84	-	-	[22]
ϕAs51	17749	Podoviridae	50-55	12	42,544	34	20	10	40-50	-	[23]
ϕA318	17749	Podoviridae	50-55	12	42,544	46	20	72	25-50	-	[23]
PVA1	Z1210	Podoviridae	50	15	41,529	21	20	108	-	-	[24]
φV141	V141	Podoviridae	66.7	11.1	43,313	48	20	55.4	-	-	[25]
vB_ValP_IME234	1651	Podoviridae	59	10	50,036	61	60	390	4-50	6-10	[26]
VEN	V2	Podoviridae	55-60	-	44,603	55	29	30	4-50		[27]
VA2001	-	Podoviridae	46 ± 5	10 ± 2	-	-	20	274	4-60	4-9	[28]
vB_ValP_IME271	1651	Podoviridae	55	-	50,345	67	90	40	<40	7-9	[29]
ΦImVa-1	17749	Podoviridae	60	-	77,479	110	15	66	27-75	5-10	[30]
vB_ValP_VA-RY-3	RZ3	Podoviridae	-	-	40,271	51	40	147	4-56	5-7	[31]
vB_ValM_R10Z	17749	Myoviridae	-	79 ± 2	247,167	111	20	45	-	-	[32]
vB_ValM_R11Z	17749	Myoviridae	-	79 ± 2	246,831	110	20	114	-	-	[32]
pVa-21	rm-8402	Myoviridae	-	-	231,998	241	70	58	-	-	[3]
VAP9	VAHN1	Myoviridae	55	65-70	82,456	-	-	-	4-60	6-8	[33]
VAP21	VAHN1	Myoviridae	65	75-80	52,636	-	-	-	4-70	7-11	[33]

ΦV208	V208	Myoviridae	70.6	129.8	45,521	76	10	87.7	-	-	[25]
φV172	V172	Myoviridae	68.4	136.8	22,378	-	10	77.8	-	-	[25]
ValKK3	17749	Myoviridae	-	-	248,088	390	-	-	-	-	[10]
VA5	10889	Siphoviridae	-	-	35,886	524	20	92.26	-20-70	2-10	[34]
vB_Va_Val-yong3	1 K04633	Siphoviridae	62 ± 4	110 ± 9.5	42,534	54	30	87.5	4-50	5-12	[12]
ValSw3-3	Va-F4	Siphoviridae	67	168	39,846	69	15	95	4-50	4-10	[4]
VAP7	V1	Siphoviridae	75	90	144,685	193	-	-	4-40	5-10	[35]
Artemius	HCMR-2 Art1	Siphoviridae	48.7 ± 0.9	107 ± 2.9	43,349	57	20	779	4-65	3-10	[8]
BUCT194	17749	Siphoviridae	58 ± 1	175 ± 1	73,099	106	<10	159	4-55	-	[30]

Table S2 The detected ORFs of vB_ValC_RH2G, including the length, RAST result, the edge-score by NCBI nucleotide database and final annotation.
Data in Excel.

References

1. Taslem Mourosi, J.; Awe, A.; Guo, W.; Batra, H.; Ganesh, H.; Wu, X.; Zhu, J. Understanding bacteriophage tail fiber interaction with host surface receptor: The key “Blueprint” for reprogramming phage host range. *Int. J. Mol. Sci.* **2022**, *23*, 12146.
2. Martha R.J., C.; Andrew M., K. Bacteriophages. *Springer.* **2009**.
3. Kim, S.G.; Jun, J.W.; Giri, S.S.; Yun, S.; Kim, H.J.; Kim, S.W.; Kang, J.W.; Han, S.J.; Jeong, D.; Park, S.C. Isolation and characterisation of pVa-21, a giant bacteriophage with anti-biofilm potential against *Vibrio alginolyticus*. *Sci. Rep.* **2019**, *9*, 6284.
4. Chen, L.; Liu, Q.; Fan, J.; Yan, T.; Zhang, H.; Yang, J.; Deng, D.; Liu, C.; Wei, T.; Ma, Y. Characterization and genomic analysis of ValSw3-3, a new *Siphoviridae* bacteriophageinfecting *Vibrio alginolyticus. J. Virol.* **2020**, *94*, e00066–20.
5. Li, Y.; Yun, H.; Chen, R.; Jiao, N.; Zheng, Q.; Yang, Y.; Zhang, R. Characterization of a vibriophage infecting pathogenic *Vibrio harveyi*. *IJMS.* **2023**, *24*, 16202.
6. Schneider, C.A.; Rasband, W.S.; Eliceiri, K.W. NIH Image to ImageJ: 25 years of image analysis. *Nat. Methods* **2012**, *9*, 671–675.
7. Yang, Y.; Cai, L.; Ma, R.; Xu, Y.; Tong, Y.; Huang, Y.; Jiao, N.; Zhang, R. A novel roseosiphophage isolated from the oligotrophic South China Sea. *Viruses* **2017**, *9*, 109.
8. Droubogiannis, S.; Pavlidi, L.; Tsertou, M.I.; Kokkari, C.; Skliros, D.; Flemetakis, E.; Katharios, P. *Vibrio* phage Artemius, a novel phage infecting *Vibrio alginolyticus*. *Pathogens* **2022**, *11*, 848.
9. Li, H.; Gao, J.; Ma, S.; Xiao, R.; Zhou, X.; Feng, W.; Zhao, S.; Luo, J.; Zhang, D. Isolation and genome sequencing of a novel lytic *Pseudoalteromonas* phage SL20. *Mar. Geonomics* **2023**, *71*, 101048.
10. Lal, T.M.; Sano, M.; Hatai, K.; Ransangan, J. Complete genome sequence of a giant *Vibrio* phage ValKK3 infecting *Vibrio alginolyticus*. *Genomics Data* **2016**, *8*, 37–38.
11. Chen, Y.; Li, W.; Shi, K.; Fang, Z.; Yang, Y.; Zhang, R. Isolation and characterization of a novel phage belonging to a new genus against *Vibrio Parahaemolyticus*. *Virol. J.* **2023**, *20*, 81.
12. Cai, R.; Li, D.; Qin, W.; Lin, W.; Pan, L.; Qian, M.; Wang, F.; Wang, C.; Mu, C.; Tong, Y. A novel *Vibrio alginolyticus* phage and its therapy application in *Portunus trituberculatus* larvae. *Aquaculture* **2024**, *579*, 740165.
13. Zerbino, D.R.; Birney, E. Velvet: Algorithms for de novo short read assembly using de bruijn graphs. *Genome res.* **2014**, 18, 821–829.
14. Garneau, J.R.; Depardieu, F.; Fortier, L.-C.; Bikard, D.; Monot, M. PhageTerm: a tool for fast and accurate determination of phage termini and packaging mechanism using next-generation sequencing Data. *Sci Rep.* **2017**, *7*, 8292.
15. Zhou, X.; Gao, J.; Xiao, R.; Qiao, Y.; Zhu, Y.; Zhang, D.; Zhang, X.; Li, H.; Xu, J. Characterization and genomic analysis of a novel *Pseudoalteromonas* phage PS_L5. *Mar. Geonomics* **2023**, *72*, 101069.
16. Altschul, S.F.; Gish, W.; Miller, W.; Myers, E.W.; Lipman, D.J. Basic local alignment search tool. *J. Mol. Biol.* **1990**, 403–410.
17. Soding, J.; Biegert, A.; Lupas, A.N. The HHpred interactive server for protein homology detection and structure prediction. *Nucleic. Acids. Res.* **2005**, *33*, 244–248.
18. Sullivan, M.J.; Petty, N.K.; Beatson, S.A. Easyfig: A genome comparison visualizer. *Bioinformatics* **2011**, *27*, 1009–1010.

19. Chan, P.P.; Lin, B.Y.; Mak, A.J.; Lowe, T.M. tRNAscan-SE 2.0: improved detection and functional classification of transfer RNA genes. *Nucleic Acids Res.* **2021**, *49*, 9077–9096.
20. Moraru, C.; Varsani, A.; Kropinski, A.M. VIRIDIC—A novel tool to calculate the intergenomic similarities of prokaryote-infecting viruses. *Viruses* **2020**, *12*, 1268.
21. Alcock, B.P.; Raphenya, A.R.; Lau, T.T.Y.; Tsang, K.K.; Bouchard, M.; Edalatmand, A.; Huynh, W.; Nguyen, A.-L.V.; Cheng, A.A.; Liu, S.; et al. CARD 2020: Antibiotic resistome surveillance with the comprehensive antibiotic resistance aatabase. *Nucleic Acids Res.* **2019**, *48*, 517–525.
22. Luo, P.; Yun, L.; Li, Y.; Tian, Y.; Liu, Q.; Huang, W.; Hu, C. Complete genomic sequence of the *Vibrio alginolyticus* bacteriophage Vp670 and characterization of the lysis-related genes, *cwlQ* and *holA*. *BMC Genomics* **2018**, *19*, 741.
23. Liu, W.; Lin, Y.-R.; Lu, M.-W.; Sung, P.-J.; Wang, W.-H.; Lin, C.-S. Genome sequences characterizing five mutations in RNA polymerase and major capsid of phages ϕA318 and ϕAs51 of *Vibrio alginolyticus* with different burst efficiencies. *BMC Genomics* **2014**, *15*, 505.
24. Zhang, J.; Cao, Z.; Xu, Y.; Li, X.; Li, H.; Wu, F.; Wang, L.; Cao, F.; Li, Z.; Li, S.; et al. Complete genomic sequence of the *Vibrio alginolyticus* lytic bacteriophage PVA1. *Arch. Virol.* **2014**, *159*, 3447–3451.
25. Fu H. Isolation, identification and genome-wide research of *Vibrio* phages from Shrimp Farm, Jimei University, **2021**.
26. Li, F.; Tian, F.; Li, J.; Li, L.; Qiao, H.; Dong, Y.; Ma, F.; Zhu, S.; Tong, Y. Isolation and characterization of a podovirus infecting the opportunist pathogen *Vibrio alginolyticus* and *Vibrio parahaemolyticus*. *Virus Res.* **2021**, *302*, 198481.
27. Kokkari, C.; Sarropoulou, E.; Bastias, R.; Mandalakis, M.; Katharios, P. Isolation and characterization of a novel bacteriophage infecting *Vibrio alginolyticus*. *Arch. Microbiol.* **2018**, *200*, 707–718.
28. Zhu Q.; Zhang J.; Wang L. Isolation and identification of a *Vibrio alginolyticus* bacteriophage Va2001 and its application. *Sci. Technol. Food Ind.* **2021**, 42(23): 102−109.
29. Li, F.; Xing, S.; Fu, K.; Zhao, S.; Liu, J.; Tong, Y.; Zhou, L. Genomic and biological characterization of the *Vibrio alginolyticus*-infecting " *Podoviridae* " bacteriophage, vB_ValP_IME271. *Virus Genes* **2019**, *55*, 218–226.
30. Tajuddin, S.; Khan, A.M.; Chong, L.C.; Wong, C.L.; Tan, J.S.; Ina-Salwany, M.Y.; Lau, H.Y.; Ho, K.L.; Mariatulqabtiah, A.R.; Tan, W.S. Genomic analysis and biological characterization of a novel *Schitoviridae* phage infecting *Vibrio alginolyticus*. *Appl. Microbiol. Biotechnol.* **2023**, *107*, 749–768.
31. Ren, Y.; Wang, L.; Chen, R.; Li, X.; Li, S.; Li, J.; Li, Q.; Wang, Z.; Xu, Y. Isolation and characterization of a novel phage vB_ValP_VA-RY-3 infecting *Vibrio alginolyticus*. *Virus Res.* **2022**, *322*, 198945.
32. Zhong, W.; Yang, Y.; Li, X.; Xu, J. Physiological and genomic characteristics of two lytic phages infecting *Vibrio alginolyticus. J Oceanogr.* **2021**, *40*, 22–34.
33. Kim, S.G.; Jun, J.W.; Giri, S.S.; Yun, S.; Kim, H.J.; Kim, S.W.; Kang, J.W.; Han, S.J.; Jeong, D.; Park, S.C. Isolation and characterisation of pVa-21, a giant bacteriophage with anti-biofilm potential against *Vibrio alginolyticus*. *Sci. Rep.* **2019**, *9*, 6284.
34. Qiao H.; Chen H.; Xu X.; Cong Yu.; Liu M.; He S.; Fei W.; Huang J.; Hu Y. Isolation, identification and biological properties of two lytic phages against *Vibrio alginolyticus*. *Journal of Fisheries of China* **2022**, 46(5): 870−884.

35. Gao, M.; Qin, Y.; Fan, H.; Zhang, X.; Li, P.; Liu, H.; Pei, G.; Mi, Z.; Xu, X.; Tong, Y.; et al. Characteristics and complete genome sequence of the virulent *Vibrio alginolyticus* phage VAP7, isolated in Hainan, China. *Arch. Virol.* **2020**, *165*, 947–953.
